# Corrigendum to: Pharmacometric dose optimization of buprenorphine in neonatal opioid withdrawal syndrome

**DOI:** 10.1111/cts.13223

**Published:** 2022-01-09

Rena Eudy‐Byrne, Nicole Zane, Susan C. Adeniyi‐Jones, Marc R. Gastonguay, Ana Ruiz‐Garcia, Gagan Kaushal, Walter K. Kraft, Clin Transl Sci. 2021;14:2171–2183. https://doi.org/10.1111/cts.13074

In the published version of the above article, the authors noticed that the surname of the sixth author was misspelled as Gagan Kushal, instead of Gagan Kaushal.

The authors regret the error.

